# Complete Coding Sequence of a Case of Chikungunya Virus Imported into Australia

**DOI:** 10.1128/genomeA.00310-17

**Published:** 2017-05-11

**Authors:** Bixing Huang, Alyssa T. Pyke, Jamie McMahon, David Warrilow

**Affiliations:** Public Health Virology Laboratory, Queensland Health Forensic and Scientific Services, Archerfield, Queensland, Australia

## Abstract

A case of chikungunya virus infection was imported from India into Australia in late 2016. Infection was diagnosed by real-time reverse transcription-PCR and confirmed by culture isolation and genome sequencing. Phylogenetic analysis of the genome sequence indicated that the virus grouped with the east/central/south African genotype.

## GENOME ANNOUNCEMENT

Infection with chikungunya virus (CHIKV) involves weeks to months of debilitating arthralgia/arthritis and can also involve myalgia, fever, headache, nausea, vomiting, and/or a rash (1, 2). Similar to dengue and Zika viruses, CHIKV has recently emerged, causing significant outbreaks with large case numbers, most notably on islands in the Indian Ocean, India, Asia-Pacific, Europe, and the Americas (2–6). It has two primary vectors: *Aedes aegypti* and *A. albopictus* (7, 8). The virus is a member of the family *Togaviridae* and the genus *Alphavirus* (9). CHIKV virions contain an 11.8-kb plus-sense single-stranded RNA genome, which encodes two open reading frames (ORFs) that are then processed into the individual viral proteins. The 5′-terminal most ORF encodes the nonstructural proteins, and utilizes an opal read-through codon to generate a full-length polyprotein. The 3′-terminal most ORF encodes the structural proteins.

A male patient with a suspected febrile illness consistent with an arthropod-borne virus (arbovirus) infection and history of recent travel to India returned to Tasmania, Australia. Real-time reverse transcription-PCR molecular analysis of an acute blood sample from the patient demonstrated the presence of CHIKV RNA, and the virus was subsequently isolated. Phylogenetic analysis (maximum likelihood model) of a PCR fragment covering 997 nucleotides (nt) of the E1 structural protein indicated that the CHIKV strain is grouped within the east/central/south African (ECSA) genotype, which is known to be currently circulating in India and is prevalent throughout other Southeast Asian regions.

On the basis of the diagnostic result, a virus genome sequence was sought. Briefly, RNA was extracted from blood using a QIAamp viral RNA extraction kit (Qiagen) without carrier RNA. Host and other contaminating microbial DNA samples were removed using a heat-labile DNase Heat & Run kit (ArcticZymes). First-strand cDNA was prepared using a ProtoScript II kit (New England Biolabs) followed by second-strand DNA synthesis using a cocktail of *Escherichia coli* DNA ligase, DNA polymerase I, and RNase H (New England Biolabs). cDNA libraries were constructed using the Nextera XT library kit and individual indices for bar coding (Illumina), following the manufacturer’s instructions. The libraries were sequenced using the V2 mid-output kit on a NextSeq 500 machine. For each sample, at least 9 × 10^6^ reads (paired at 2 × 151 nt) were obtained, and the output was filtered to remove human sequence data. Assembly was performed by mapping reads to a reference genome (DQ443544) using Geneious R8 software (10) at the lowest sensitivity settings.

The assembled genome is a coding-complete draft. The putative ORFs were a nonstructural polyprotein of either 1,856 amino acids (aa) (nsP1-nsP2-nsP3) or 2,474 aa (nsP1-nsP2-nsP3-nsP4) for the termination or read-through product, respectively, and a structural polyprotein of 1,248-aa (C-E3-E2-6K-E1). It had a 99.3% nucleotide sequence identity with an isolate from a Réunion Island outbreak but, interestingly, was consistent with other recently reported Indian CHIKV strains (GenBank accession nos. EU564335 and EF451148) and did not contain the A226V E1 mutation implicated with the Réunion Island epidemic.

### Accession number(s).

The genome sequence was deposited in GenBank under accession number KY751908.
